# Effect of nintedanib on non‐small cell lung cancer in a patient with idiopathic pulmonary fibrosis: A case report and literature review

**DOI:** 10.1111/1759-7714.13437

**Published:** 2020-04-14

**Authors:** Toshihiro Shiratori, Hisashi Tanaka, Chiori Tabe, Junichiro Tsuchiya, Yoshiko Ishioka, Masamichi Itoga, Kageaki Taima, Shingo Takanashi, Sadatomo Tasaka

**Affiliations:** ^1^ Department of Respiratory Medicine Hirosaki University School of Medicine Graduate School of Medicine Hirosaki Japan; ^2^ Health Administration Center Hirosaki University Hirosaki Japan

**Keywords:** Idiopathic pulmonary fibrosis, lung cancer, nintedanib

## Abstract

Nintedanib has been approved for the treatment of idiopathic pulmonary fibrosis (IPF). In addition, in EU countries, nintedanib plus docetaxel is used for patients with advanced non‐small cell lung cancer (NSCLC) after first‐line chemotherapy. Here, we report a case of advanced NSCLC in a patient with IPF successfully treated with nintedanib monotherapy. A 69‐year‐old man was diagnosed with NSCLC complicated by IPF. After three lines of chemotherapy, he still had progressive disease. Because his IPF had also progressed, requiring supplemental oxygen, we decided to start best supportive care and introduced nintedanib to treat his IPF. One month later, we observed a partial remission of the primary tumor and pleural disseminations without severe adverse events. Nintedanib monotherapy might therefore be an effective therapeutic choice for NSCLC in patients with IPF who are unable to tolerate cytotoxic chemotherapy.

**Key points:**

- Efficacy of nintedanib administered in a NSCLC patient with IPF.
- Nintedanib monotherapy might be a therapeutic option for NSCLC patients with IPF who are unable to tolerate chemotherapy.

## Introduction

Idiopathic pulmonary fibrosis (IPF) is a risk factor for lung cancer development,^1^ and IPF is observed in 5.8% to 15.2% of patients with lung cancer at diagnosis.^2^ Because patients with IPF have been excluded from most clinical trials of lung cancer therapies, the optimal chemotherapy for such patients remains to be established. There are only a few prospective phase II studies, which have evaluated the safety and efficacy for patients with non‐small cell lung cancer (NSCLC) and interstitial lung disease (ILD).^3^, ^4^, ^5^, ^6^ Nintedanib has been shown to suppress pulmonary function decline and reduce the risk of acute exacerbation in IPF patients.^7^ Moreover, nintedanib plus docetaxel has been shown to be effective in patients with advanced NSCLC.^8^

## Case report

A 69‐year‐old man was diagnosed as NSCLC (cT2aN2M0, stage IIIA) in March 2017. He had a smoking history and no special family history. Because high‐resolution computed tomography (HRCT) scan showed honeycombing in the lung bases, suggesting IPF, we chose carboplatin plus pemetrexed in combination with bevacizumab as the first‐line treatment. Thereafter, docetaxel plus ramucirumab were administered as the second‐line and carboplatin plus S‐1 as the third‐line treatments. After these chemotherapies, we observed progress of IPF and subsequently introduced supplemental oxygen therapy. Because of the progression of NSCLC and no more regimen with safety in a patient with IPF, we decided to start best supportive care and initiated treatment with nintedanib for IPF in May 2018.

**Figure 1:**
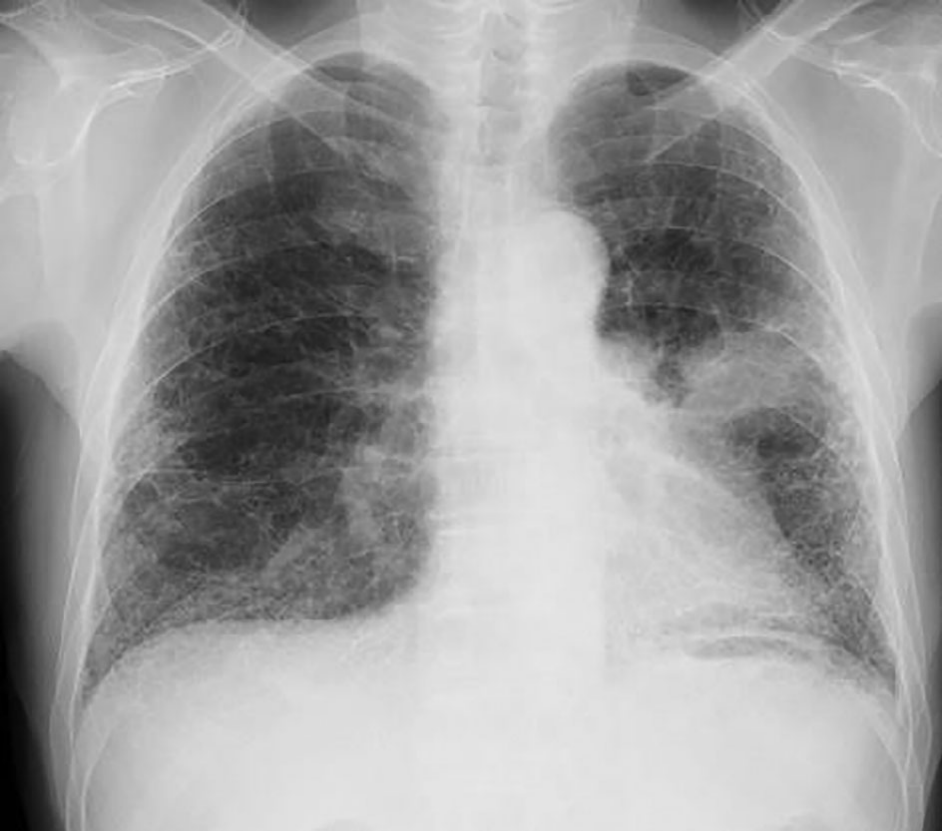
Chest radiography on the initiation of nintedanib showed reticular shadows in both lung fields and a mass lesion in the left middle lung field.

**Figure 2:**
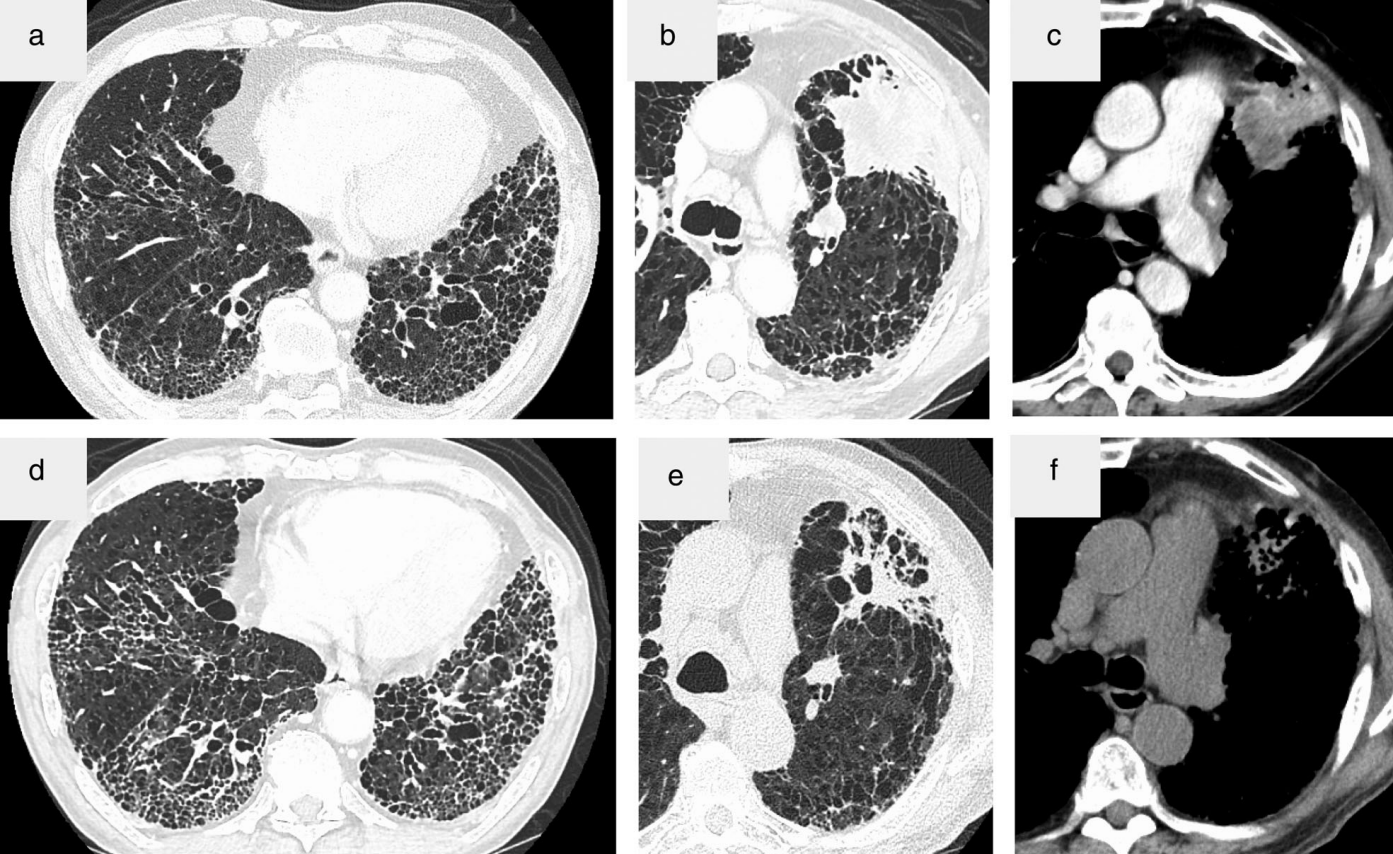
(**a**) Chest computed tomography (CT) scan at initial referral to our hospital showed honeycomb lung, traction bronchiectasis in the dorsal side of both lung bases and interstitial lung disease of usual interstitial pneumonia pattern. (**b**) CT scan on the initiation of nintedanib showed a solid mass measuring 50 mm in diameter in the left upper lobe, (**c**) with pleural dissemination and (**d**) that the interstitial pneumonia was getting worse. (**e**) CT scan taken one month after the initiation of nintedanib showed regression of the primary lesion and (**f**) pleural disseminated lesions in the left upper lobe.

On the first day of nintedanib at 150 mg twice a day, his vital signs were normal except for tachypnea. Chest auscultation revealed fine crackles in both lower lung fields and clubbed fingers were also observed. A chest radiography showed reticular shadows in bilateral lung fields and a mass shadow in the left middle lung (Fig 1). Chest computed tomography (CT) scan at initial referral to our hospital, one year before the initiation of nintedanib, showed honeycomb lung, traction bronchiectasis in the dorsal side of both lung bases and interstitial lung disease of usual interstitial pneumonia (UIP) pattern (Fig 2a). Chest CT showed a tumor in the left upper lobe with pleural disseminated and his UIP was getting progressively worse (Fig 2b–d). The serum level of carcinoembryonic antigen (CEA) was as high as 60.1 ng/mL and sialylated carbohydrate antigen Krebs von den Lungen‐6 (KL‐6) level was elevated to 2780 U/mL. On spirometry, the forced vital capacity (FVC) was 2.75 L, which was 0.48 L lower than a year before. He also had impaired diffusing capacity with DLCO of 14.5% (Table 1). After the introduction of nintedanib, we observed modest elevation of liver enzymes, which improved after the short‐term administration of ursodeoxycholic acid.

**Table 1:** Laboratory findings

Hematology			Biochemistry			Serology			Pulmonary function		
WBC	5300	μL	TP	7	g/dL	CRP	1.41	mg/dL	FVC	2.75	L
Neu	47	%	Alb	2.5	g/dL	KL‐6	2780	U/mL	FEV 1.0	2.39	L
Lym	33	%	T‐Bil	0.3	g/dL	SP‐D	184	ng/mL	%FEV 1.0	83.3	%
Mono	12	%	AST	34	IU/L				FEV 1.0%	86.9	%
Eos	5	%	ALT	31	IU/L	Tumor marker			DLCO	14.5	%
Baso	1	%	LDH	260	IU/L	CEA	60.1	g/mL	%VC	79.1	%
RBC	2.51	x 10^6^/μL	BUN	8	g/dL	CYFRA	3.1	g/mL			
Hb	10.4	g/dL	Cre	0.77	g/dL						
PLT	19.6	x 10^4^/μL	Na	138	Eq/L	Blood gas analysis (2 L nasal cannula oxygen)			6 minutes walk test (2 L nasal cannula oxygen)		
			K	4	Eq/L						
			Cl	106	Eq/L	pH	7.447		Walking distance	85	m
			Ca	8.9	g/dL	pO2	71.2	mHg	Lowest SpO_2_	84	%
						pCO2	35.9	mHg			
						HCO3	24.4	ml/L			

One month later, chest CT revealed regression of the primary tumor and pleural dissemination (Fig 2e,f). The CEA level lowered to 12.8 ng/mL. Although he did not experience exacerbation of IPF, he was readmitted because of pleural effusion and respiratory failure and subsequently died of the cancer progression.

## Discussion

In the present case, we introduced nintedanib monotherapy in an IPF patient with NSCLC, and seven months of disease control was achieved with a modest adverse event, but no exacerbation of IPF. Nintedanib is an indolinone derivative that potently blocks the proangiogenic pathways mediated by vascular endothelial growth factor (VEGF) receptors 1–3, platelet‐derived growth factor (PDGF) receptors and fibroblast growth factor (FGF) receptors.^9^ In a preclinical study, nintedanib demonstrated potent antitumor effects in xenograft models of human lung cancer.^10^ Previous clinical trials on nintedanib for NSCLC are summarized in Table 2. In a phase II study, which assessed the efficacy, safety, and tolerability of nintedanib in stage IIIB/IV NSCLC, the 73 patients recruited tolerated the continuous treatment and had no significant difference in efficacy between treatment arms (nintedanib 250 mg twice daily vs. 150 mg twice daily).^11^ The median progression‐free survival (PFS) was 6.9 weeks and the median overall survival (OS) was 21.9 weeks with no significant difference between the two groups.^11^ In another study, nintedanib (200 mg b.i.d.) in combination with docetaxel (75 mg/m^2^) significantly prolonged both PFS and OS, compared with placebo plus docetaxel.^8^ Hanna and colleagues compared efficacy and safety between nintedanib (200 mg b.i.d.) plus pemetrexed (500 mg/m^2^) and pemetrexed alone in patients with advanced non‐squamous NSCLC.^12^ Although nintedanib plus pemetrexed achieved significantly longer PFS, there was no difference in OS between the two arms.^12^ Because these clinical trials did not include patients with IPF, the safety and efficacy of nintedanib in NSCLC patients with IPF remains unclear. To the best of our knowledge, there has been only one case report published on nintedanib monotherapy for a patient with NSCLC and IPF. Fukunaga and colleagues reported a case of squamous cell lung cancer in a patient with IPF in whom nintedanib prevented the progression of IPF and mentioned the possibility that nintedanib might reduce lung cancer incidence in patients with IPF or prolong the survival time.^13^ Further investigation is needed to evaluate the efficacy of nintedanib monotherapy for patients with NSCLC and IPF. Nintedanib monotherapy might therefore be effective for some patients with NSCLC and IPF who are unable to tolerate cytotoxic chemotherapy.

**Table 2:** Nintedanib phase II to phase III clinical trials

Phase	Reference	Patient characteristics	*n*	Treatment	ORR (%)	Median PFS	Median OS
III	Reck *et al*.^8^	NSCLC second‐line	1314	Docetaxel + Nintedanib 200 mg/b.i.d. vs. Docetaxel	NE	3.4 vs. 2.7 months	10.1 vs. 9.1 months
II	Reck *et al*.^11^	NSCLC second‐ or third‐line	73	Nintedanib 250 mg/b.i.d. or 150 mg/b.i.d.	NE	6.9 weeks	21.9 weeks
III	Hanna *et al*.^12^	Nonsquamous NSCLC previously treated	713	Pemetrexed + Nintedanib 200 mg/b.i.d. vs. Pemetrexed	9.1 vs. 8.3	4.4 vs. 3.6 months	12.0 vs. 12.7 months

NE, not evaluated; NSCLC, non‐small cell lung cancer; ORR, overall response rate; OS, overall survival; PFS, progression‐free survival; Sq, squamous.

## Disclosure

All authors declare they have no competing interests.
